# Primary cutaneous adenoid cystic carcinoma of the scalp: A case report, immunohistochemistry and review of the literature

**DOI:** 10.1002/ski2.118

**Published:** 2022-04-28

**Authors:** Bhakinai Temnithikul, Suthat Rungrunanghiranya, Piyakan Limtanyakul, Suthep Jerasuthat, David G. Paige

**Affiliations:** ^1^ Department of Internal Medicine, Faculty of Medicine Srinakharinwirot University Bangkok Thailand; ^2^ Department of Dermatology Chulabhorn Royal Academy Bangkok Thailand; ^3^ Department of Internal Medicine, Faculty of Medicine Ramathibodi Hospital Mahidol University Bangkok Thailand; ^4^ Barts & The London NHS Trust The Royal London Hospital Whitechapel London UK

## Abstract

Primary cutaneous adenoid cystic carcinoma (PCACC) is an uncommon adnexal skin tumour with fewer than 200 cases studied in detail in the English literature. We describe the diagnosis and treatment of a few Southeast Asian cases of PCACC on the scalp of a 70‐year‐old Thai female. She presented with a slow‐growing, painless, solid to cystic, skin‐coloured tumour on her scalp. When excisional biopsy was done, histopathological findings showed dermal tumour that had a classic histologic appearance composed of basaloid cells arranged in a cribriform pattern with ‘punched‐out’ pseudocysts filled with mucin (swiss cheese pattern) and had perineural invasion. The clinical and histopathological findings, and complete investigations confirmed the diagnosis of PCACC. Our case illustrates that PCACC, is an important histopathological differential diagnosis to bear in mind due to its locally aggressive nature and tendency to recur due to perineural invasion. A wide local excision with at least 2 cm of tumour‐free margins was performed, which revealed no residual carcinoma. The patient remained disease‐free for 16 months after diagnosis. PCACC is usually located on the head or neck of people in their sixth decade of life, with a female predominance. The aetiology of PCACC is unclear. The majority of PCACCs have the *MYB‐NFIB* fusion gene or show overexpression of *MYB* by immunohistochemistry. Diagnosis of PCACC is primarily based on the characteristic histological appearance, as there are no distinguishing clinical features. The diagnosis of PCACC requires careful exclusion of infiltration or metastasis from other primary lesions. Treatment of this rare tumour is wide surgical excision with at least 2 cm of tumour‐free margins to reduce the risk of local recurrence, and long‐term follow‐up for possible recurrence of PCACC is recommended. This case emphasizes the importance of careful inspection for the diagnosis of PCACC after initial surgery and pathological evaluation of the mass lesion for appropriate diagnosis and therapy.

## Graphical abstract

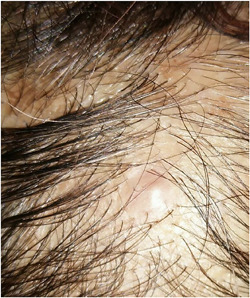

1

**What is already known about this topic?**

- Solitary skin colour tumour on the scalp represents a diagnostic challenge for clinicians and diagnosis is primarily based on characteristics of histological features.

**What does this study add?**

- To decrease incidence of local recurrence which perineural invasion plays a role, wide surgical excision with at least 2 cm of tumour‐free margins is recommended.
- There was a slightly better prognosis in male, under 50 years, localized diseases, located on face, head and neck region.

## 2 CASE REPORT

A 70‐year‐old Asian woman presented with isolated painful scalp papule on left lateral posterior of her scalp for 1‐year duration, which had grown progressively. She denied any history of trauma, pruritus or bleeding in the area and had no history of weight loss, fever or fatigue. She had no known underlying disease and no history of skin tumours in her family. The size of the papule gradually increased in size and became more painful over the next month.

**FIGURE 1:**
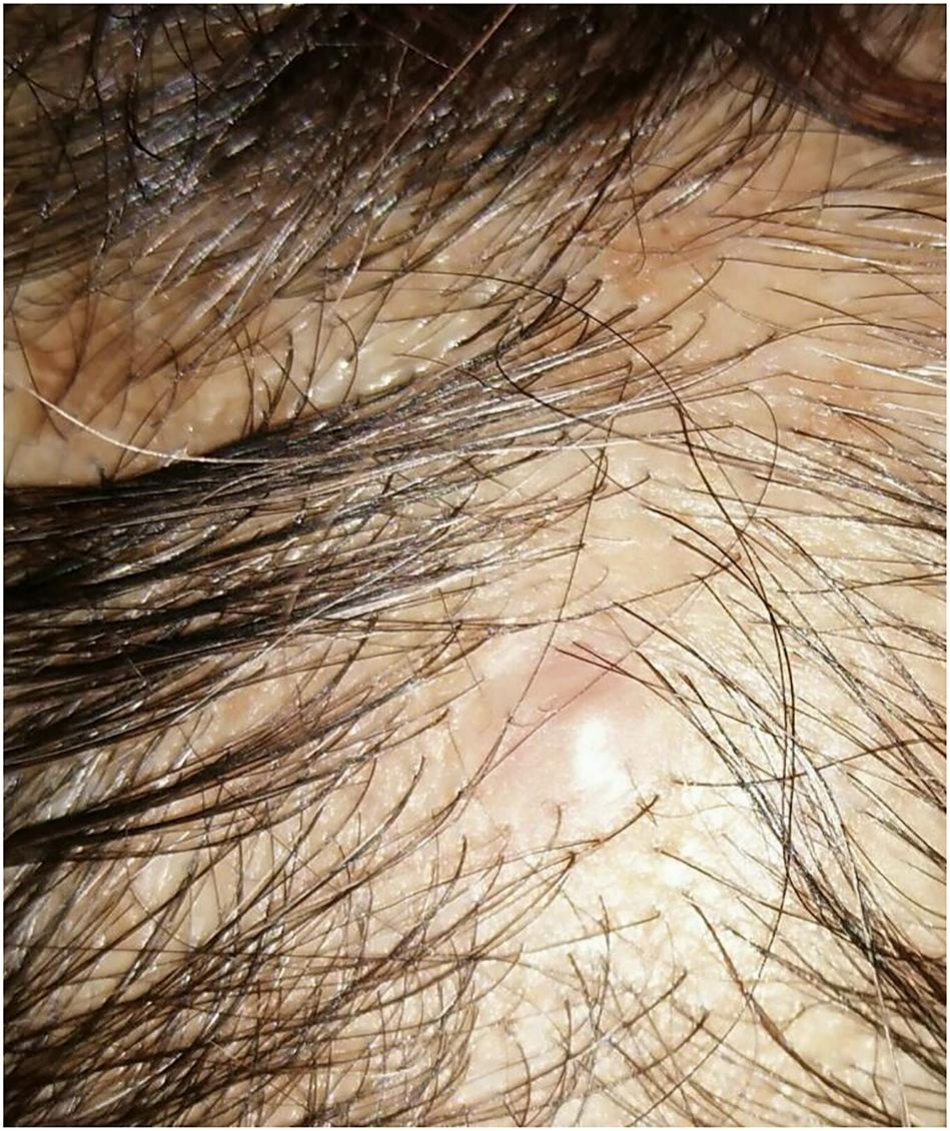
Solitary, firm, tender, 5‐mm, flesh‐coloured papule on the left upper lateral posterior aspect of her scalp

Physical examination revealed a solitary, firm, tender, 5‐mm, flesh‐coloured papule on left lateral posterior of her scalp (Figure 1). The rest of her cutaneous exam was normal. No lymphadenopathy was presented. Our initial clinical differential diagnosis was pilomatrixoma (a clinically lookalike tent sign), adnexal tumours and nodular basal cell carcinoma.

**FIGURE 2:**
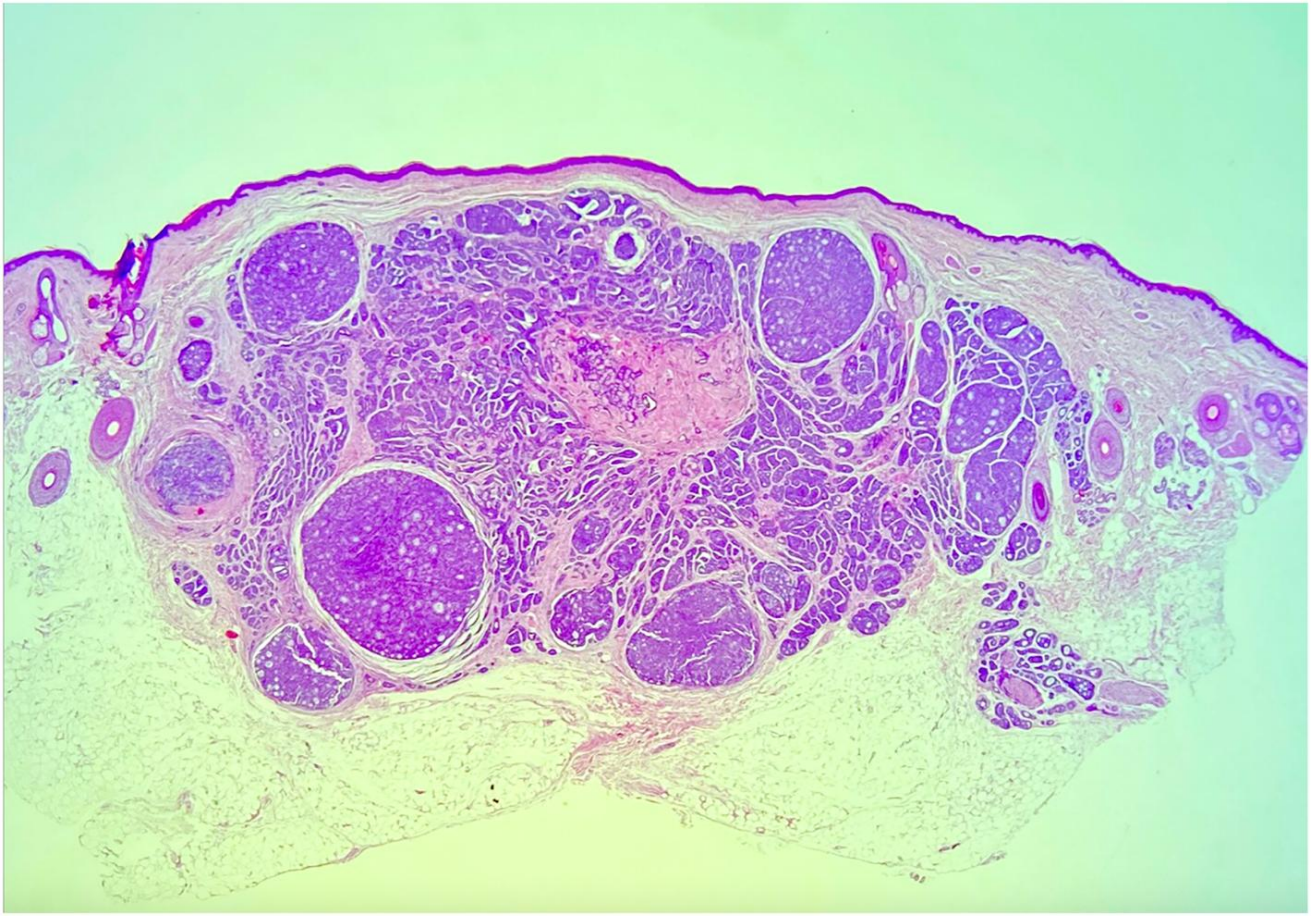
A well‐defined deep dermal nodule (H&E, × 4)

**FIGURE 3:**
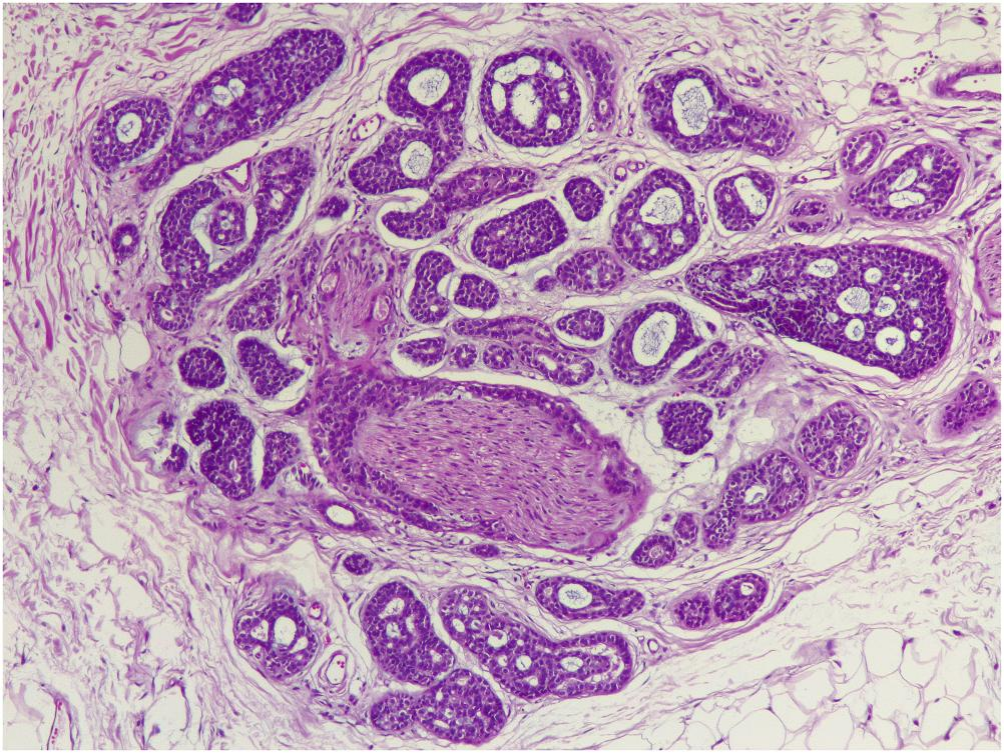
Composed of basophilic epithelial component forming ductal structure and cribriform pattern (swiss cheese pattern) with perineural invasion (H&E, × 100)

**FIGURE 4:**
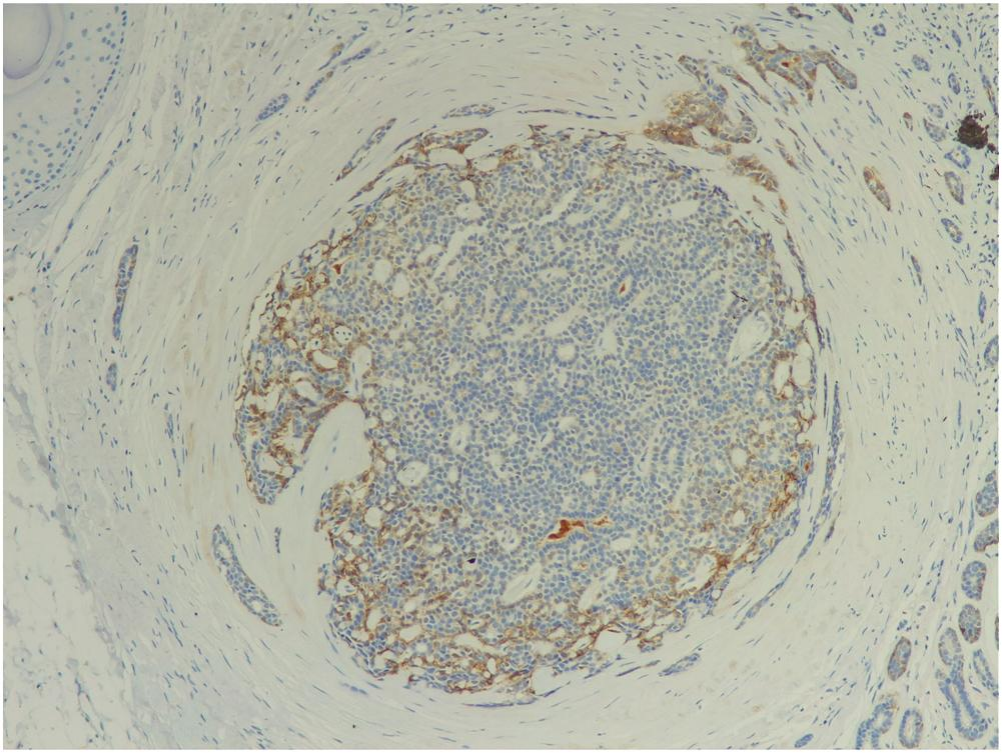
The immunohistochemical stains proved positive for CEA (×100)

The lesion was surgically excised and histopathology revealed an adenoid cystic carcinoma (ACC) and perineural invasion. The immunohistochemical stains proved positive for carcinoembryonic antigen (CEA) (Figures 2, 3, 4). The resection margins were not tumour‐free. A further wide surgical excision with 2 cm margins was carried out.

A thorough examination of the ears, nose and throat was performed, which was unremarkable. Computed tomography scan and magnetic resonance imaging were done of the paranasal sinus regions, maxillomandibular region, neck and thorax. Both investigations were unremarkable, supporting a cutaneous origin for this tumour. The diagnosis is compatible with primary cutaneous adenoid cystic carcinoma (PCACC).

The wide excision specimen showed no residual tumour. The patient remains on six‐monthly follow‐up and, to date, shows no signs of recurrence.

## 3 DISCUSSION

ACC is a very rare, homogeneous subtype of the well‐defined group of malignant sweat gland tumours.^1^ ACC originates from secretory glands, most commonly from major and minor salivary glands, which can be found at other sites such as excretory glands of genital tract, lacrimal glands and ceruminous glands.

PCACC, a rare tumour(<1% of all head and neck cancers) first described by Boggio^2^ in 1975, is classified as an adnexal skin tumour by the World Health Organization.^3^ PCACC typically manifests as slow‐growing, rarely ulcerated, painless, solid to cystic, skin‐coloured tumours 0.5–8.0 cm in diameter on the head, face, breast, external auditory canal, upper thorax, major bronchi, uterine cervix, upper extremities and skin.^4^ The scalp (at least 40%) and chest have been the sites of predilection.^5^ When diagnosing PCACC, it's important to rule out the possibility of metastatic disease to the skin from another primary locations, and several primary skin tumours, including the adenoid‐cystic variant of basal cell carcinoma, mucinous carcinoma, dermal cylindroma, spiradenoma, pilomatricoma and the primary cutaneous cribriform apocrine carcinoma.

PCACC is a low‐grade inactive malignancy with a favourable prognosis compared with salivary ACC, which shows more aggressive behaviour. PCACC has a high degree of local invasiveness, with the majority of cases remaining confined to the skin, but only a few cases of distant metastases (usually to the lung and regional lymph nodes) have been reported.^5^ Metastasis may develop lately up to 18 years in one case report.^6^ Perineural invasion is common, occurring in 76% of PCACC patients and is associated with a 46% recurrence rate compared to 22% in patients without perineural invasion.^7^ PCACC appears to be linked to subsequent lymphohematologic malignancy and autoimmune diseases such as Rheumatoid arthritis,^7^ Hashimoto thyroiditis,^8^ Sjögren syndrome and papillary thyroid cancers. When ACC occurs in the scalp, alopecia is a common symptom.^4^

There are approximately 200 reported cases in the current literature.^9^, ^10^ PCACC often occurs in adults with an average age in their sixth decade of life (mean age: 58 years) and female predominance (3:2).^7^ According to population‐based study in the United States, of 152 PCACC patients diagnosed during the 30‐year period, the overall PCACC incidence ratio was 0.23 per 1 million person‐years.^10^

The aetiology of PCACC is unclear. They are probably driven by somatic mutations. However, somatic mutation patterns are unexplored.^11^ This means that a lot of PCACC had the fusion gene *MYB‐NFIB* or showed high levels of *MYB* by immunohistochemistry, which suggests that salivary and PCACC share a common development process.^12^

Diagnosis of PCACC is primarily based on characteristics of histological features because there are no distinguishing clinical features of the tumour. PCACC has been misinterpreted as having metastatic lesions. As a result, a full clinical and radiological examination needs to be done to look for signs of primary disease elsewhere, especially in the salivary glands.

PCACC is a dermal tumour that is not connected to the epidermis. It has a classic histologic appearance and is composed of dermal islands of basaloid cells arranged in a cribriform pattern with ‘punched‐out’ pseudocysts filled with mucin (swiss cheese pattern). Tumour cells are basaloid with a number of mitotic figures, no true palisading and a tendency to show perineural infiltrates, particularly at the periphery, which is characteristic for this tumour. From viewpoint of histopathology, microscopic differential diagnoses include basal cell carcinoma with adenoid cystic differentiation, cylindroma, mucinous apocrine carcinoma and apocrine mixed tumour of skin and metastasis arising from other malignancies with histological features of ACCs.^7^ All the mentioned lesions closely mimic the PCACC at histology.

True luminal spaces are surrounded by modified myoepithelial cells that are periodic acid‐Schiff‐positive and diastase‐resistant. The mucin is sialomucin, detected by an Alcian blue stain (pH 2.5).^9^ Although there is significant overlap in the histopathology of primary cutaneous and salivary gland ACC, but different characteristic immunohistochemical profiles can be distinguished between them.

Immunohistochemistry is characteristic but not diagnostic. Immunohistochemical study of the tumour shows two differentiated patterns, epithelial/ductal lines and myoepithelial line.^9^ ACC is immunoreactive for CEA, epithelial membrane antigen, AE1/AE3 and amylase. ACC is positive for cytokeratins (including CK7), CD117 (cKIT), CK15 and SOX‐10.^13^ PCACC is positive for CK15 and CK7. CK15 has low sensitivity and high specificity, on the other hand, CK7 lacks specificity.^12^ Myoepithelial cells in ACC stained for typical myoepithelial markers, including smooth muscle actin, S100 and p63.^9^, ^12^ The aggregates often stain with CK7 in centre with a small layer of actin‐positive myoepithelial cells at the periphery. The presence of type IV collagen and laminin in cystic spaces suggests the presence of basement membrane material.^9^ Parts of the tumour may be positive for BerEP4, S100 and cKit.^1^

PCACC is a locally invasive cancer; 70% of 130 PCACC cases had a localized stage, 25% had regional metastasis and 5% had distant metastasis.^10^ Within 58 months of follow‐up, 44% of cases recurred.^7^ The overall 5‐year survival rate was 96.1%.^10^ There was a slightly better prognosis (in terms of 5‐year‐survival) in male patients, patients aged under 50 years, patients with localized diseases and tumours located on face, head and neck region (rather than on the trunk).^10^

Wide surgical excision with at least 2 cm tumour‐free margins is advised to reduce the risk of local recurrence, and long‐term follow‐up for recurrence is recommended.^9^ Mohs micrographic surgery has been used in many cases, with no cases of local recurrence during the follow‐up period.^14^ Currently, routine lymph node dissection is not advised unless there is evidence of lymphadenopathy.^7^ Radiotherapy and chemotherapy can be used as adjuvant treatments, but they have shown variable success. If patients deemed unable to tolerate surgical resection or have local recurrent tumours, palliative radiotherapy has also been used. Chemotherapy is commonly used to treat metastatic tumours. The successful treatment of PCACC with pulmonary metastases with a combination therapy (doxorubicin with cyclophosphamide and prednisolone or cisplatin) has been reported.

In conclusion, we described a few cases of scalp PCACC in Southeast Asia. PCACC is a rare malignancy that should be considered in the differential diagnosis of adnexal neoplasms and, when occurring on the head and neck, must be distinguished from cutaneous involvement by salivary ACC and requires careful exclusion of infiltration or metastasis from other primary lesions. PCACC is divided into two sections histologically: ductal and myoepithelial (like ‘punched‐out’ pseudocysts filled with mucin in a ‘swiss cheese’ pattern). Treatment of this rare tumour is wide surgical excision with at least 2 cm of tumour‐free margins to reduce the risk of local recurrence in which perineural invasion plays a role after resection, and long‐term follow‐up for possible recurrence of PCACC is recommended.

## CONFLICT OF INTEREST

The authors declare no conflict of interest.

## AUTHOR CONTRIBUTIONS

**Bhakinai Temnithikul:** Conceptualization (lead); Data curation (lead); Formal analysis (lead); Project administration (lead); Resources (lead); Writing – original draft (lead); writing – review & editing (lead). **Suthat Rungrunanghiranya:** Conceptualization (support); Formal analysis (support); Project administration (support). **Piyakan Limtanyakul:** Conceptualization (equal); Data curation (equal); Formal analysis (support); Supervision (equal); Project administration (equal); Resources (equal); Writing – original draft (support); writing – review & editing (support). **Suthep Jerasuthat:** Conceptualization (support); Data curation (support); Project administration (support); Resources (support). **David G. Paige:** Conceptualization (support); Data curation (support); Writing – original draft (support); writing – review & editing (support).

## Data Availability

Data is openly available in a public repository that issues datasets with DOIs.
